# How Overlimiting Current Condition Influences Lactic Acid Recovery and Demineralization by Electrodialysis with Nanofiltration Membrane: Comparison with Conventional Electrodialysis

**DOI:** 10.3390/membranes10060113

**Published:** 2020-05-27

**Authors:** Marielle Beaulieu, Véronique Perreault, Sergey Mikhaylin, Laurent Bazinet

**Affiliations:** 1 Institute of Nutrition and Functional Food (INAF) and Department of Food Sciences, Pavillon Paul-Comtois, Université Laval, Quebec, QC G1V 0A6, Canada; marielle.beaulieu.2@ulaval.ca (M.B.); veronique.perreault.5@ulaval.ca (V.P.); Sergey.Mikhaylin@fsaa.ulaval.ca (S.M.); 2 Laboratoire de Transformation Alimentaire et Procédés Électro-membranaires (LTAPEM, Laboratory of Food Processing and Electro-Membrane Processes), Pavillon Paul Comtois, Université Laval, Quebec, QC, G1V 0A6, Canada

**Keywords:** acid whey, lactic acid recovery, demineralization, electrodialysis, energy consumption, nanofiltration membrane

## Abstract

Acid whey is the main co-product resulting from the production of fresh cheeses and Greek-type yogurts. It generally goes through a spray-drying process prior to valorization, but it needs to be deacidified (lactic acid recovery) and demineralized beforehand to obtain a powder of quality with all the preserved compounds of interest such as lactose and proteins. Electrodialysis (ED) is a process actually used for acid whey treatment, but scaling formation at the surface of the ion-exchange membrane is still a major problem. In this work, a combination of two new avenues of ED treatment has been studied. First, the integration of a nanofiltration (NF) membrane in an ED conventional stack was compared to a classical ED stack with an anion-exchange membrane in a standard current condition. Secondly, both configurations were tested in the overlimiting current condition to study the impact of electroconvective vortices on process efficiency. The combined effects of the NF membrane and overlimiting current condition led to a higher lactic acid recovery rate of acid whey (40%), while the conventional ED stack in the overlimiting current condition led to a higher demineralization (87% based on the total cation concentration). Those effects were related to the conductivity, pH, global resistance, and energy consumption of each treatment that are influenced by water splitting phenomenon, which was decreased in the overlimiting condition.

## 1. Introduction

Dairy production generates effluents with variable composition depending on the final product. Acid whey is generated from the manufacture of Greek-type yoghurts, fresh cheeses (cream or cottage cheeses), and caseins resulting from milk acidification. In the past few years, the demand for those dairy products has continued to increase, leading to the production of significant volumes of acid whey. Considered as the most contaminated waste generated in dairy production because of its high organic content [1,2], this co-product can be valorized in different ways to limit its environmental impact [2]. Since whey contains compounds of interest, such as lactose and whey proteins, with high nutritional value [3,4], it is processed into powder by spray drying [5] to facilitate its use and transportation. However, the high calcium and lactic acid contents in acid whey impact the quality of the powder generated and the energy consumption of the drying process [6,7]. In fact, as the concentrations of calcium and lactic acid in whey increase, the glass transition temperature of lactose decreases, affecting its properties [6,7]. Consequently, lactose is mainly present in crystalline phase, leading to a sticky powder that reduces process efficiency. Considering those facts, acid whey needs to be demineralized and deacidified to optimize the drying process and limit wastes [5]. 

Many processes and experimental conditions have been tested to realize this critical preliminarily step such as nanofiltration (NF) [8], ion-exchange resin [9] and ion-exchange resin coupled to electrodialysis (ED) [10]. However, NF allows partial demineralization and deacidification of acid whey (46–60% demineralization and 30% reduction in lactic acid) [11], and ion-exchange resin produces a large amount of effluents during regeneration, which needs to be treated. Consequently, ED without coupling ion-exchange resins would be a very interesting alternative in terms of ecoefficiency, since ED is able to separate simultaneously minerals and organic acid anions [9]. However, scaling (mineral fouling) formation at the surface of the ion-exchange membrane is still a major problem that needs to be solved to preserve membrane integrity and optimize the valorization of acid whey. Different solutions are already available to diminish or control ED scaling to some extent, such as pre-treatment of the feed solution [12,13], the control of hydrodynamic conditions [14,15], reversal ED [16] and modifications of the membrane surface properties [14,17]. However, a cleaning-in-place procedure, which employs chemicals and generates effluents, is still needed to recover the process performance over extended periods [18]. Recent investigations showed that an overlimiting current condition improved ED performances [19,20]. Indeed, in overlimiting conditions, the electroconvective vortices (EVs) generated facilitate the transport of ions toward the ion-exchange membrane (IEM) surface and limit fouling formation. Hence, Bukhovets et al. [21] reported the influence of electroconvection on organic fouling prevention by the “washing out” effect of EVs, while Mikhaylin et al. [22] demonstrated the positive effect of electroconvection on IEM scaling mitigation. To the best of our knowledge, the application of overlimiting current condition has only been tested on the demineralization of model solution with calcium and magnesium compounds [22] and on whey protein concentrate [23]. No information is available in the literature concerning the influence of this non-conventional current condition on the simultaneous acid recovery and demineralization of complex solutions such as acid whey.

In addition, the use of charged filtration membranes stacked in an ED cell was demonstrated to be effective for demineralization of solutions. Hence, in 2011, Bazinet and Moalic [24] were the first to use a nanofiltration membrane (NF) in an ED stack for the demineralization of sea water. They demonstrated that the use of NF membrane can also allow the selective separation of cations. More recently, Ge et al. [25] exemplified such a selective cation fractionation of EDNF with H^+^/Zn^2+^ and Na^+^/Mg^2+^ systems and demonstrated that an NF membrane can increase the limited current density in ED. In the case of acid whey demineralization and the deacidification process, these membranes could allow a better mass transfer due to their larger pore size and their surface charge, particularly for lactic acid. However, the use of nanofiltration membrane in an ED cell for the deacidification of acid whey has never been reported in the literature.

In this context, the main goal of this study was to explore the use of NF membrane in the replacement of anion-exchange membrane (AEM) stacked in the ED cell as well a combination of the overlimiting current condition in order to potentially optimize lactic acid recovery and the demineralization of acid whey and prevent the formation of scaling.

## 2. Materials and Methods

### 2.1. Whey

The acid whey samples were obtained from the Parmalat Canada (Winchester, Ontario, Canada) dairy processing plant and transported at 4 °C until they reached the laboratory. They were aliquoted, stored at −30 °C, and thawed at 4 °C before each experiment. Their composition, as described in Table 1, was in accordance with the literature [6,26,27].

### 2.2. Electrodialysis Cell

**Figure 1:**
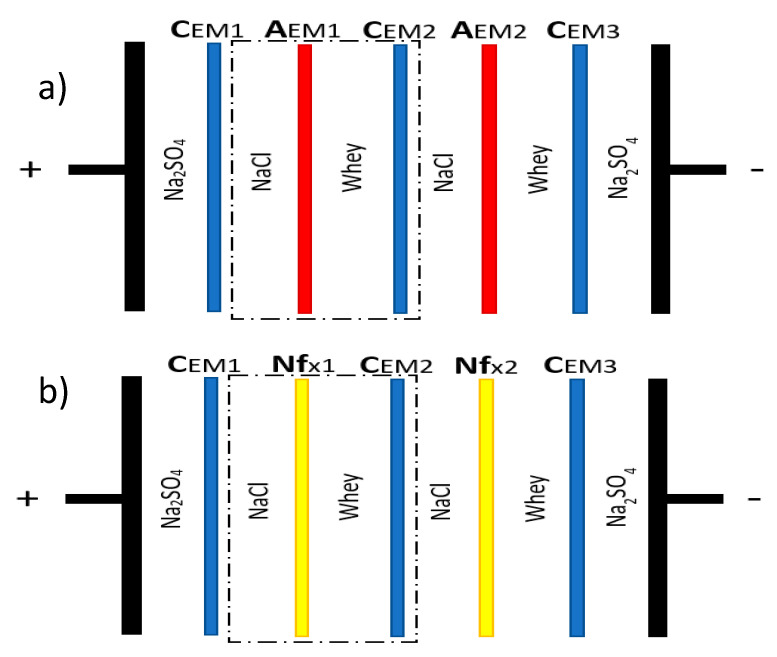
ED cell configuration (**a**) “CACAC” and (**b**) “CNfCNfC” used for acid whey deacidification and demineralization.

An MP-type ED cell (ElectroCell AB, Täby, Sweden) with an effective membrane surface area of 100 cm^2^ was used for the experimentations in this work. This cell included a dimensionally stable electrode (DSA-O_2_) as the anode and a stainless steel electrode as the cathode. The first membrane configuration “CACAC” (Figure 1a), a conventional industrial ED configuration for demineralization, combined three cation-exchange membranes (CMX-fg, Astom, Tokyo, Japan) and two anion-exchange membranes (AMX-fg, Astom) as described by Chen et al. [26] and Dufton et al. [27]. In the second configuration “CNfCNfC” (Figure 1b), the AEMs were replaced by NF membranes with a molecular weight cut-off of 500 Da (NFX, Synder Filtration, Vacaville, USA) to obtain a similar configuration to the one described by Bazinet and Moalic [24]. According to the manufacturer information, the NF membranes were positively charged at acid whey’s pH.

NaCl 5.5 g/L (Fisher Scientific, Ottawa, ON, Canada) was used as the recovery solution and Na_2_S0_4_ 20.0 g/L (Anachemia, VWR International, Mississauga, ON, Canada) was used as the electrode rinsing solution. First, 500 mL of whey and NaCl solutions circulated in two different loops to create two deacidification/demineralization units (dotted line, Figure 1a,b). In underlimiting current density ED experiments, a 15A/60V power supply (Model 9110, BK Precision, Yorba Linda, CA, USA) was used to generate the potential difference, while a 5A/100 V power supply (Ionics Inc, Watertown, MA, USA) was used in the overlimiting current condition.

### 2.3. Protocol

**Figure 2:**
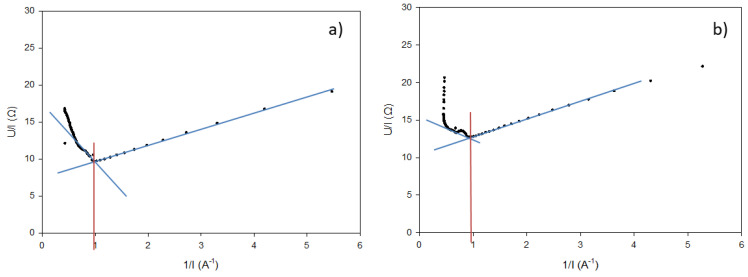
Determination of limiting current density (LCD) of (**a**) CACAC and (**b**) CNfCNfC membrane configuration.

Prior to ED experiments, membrane samples were characterized in terms of electrochemical characteristics (current density, resistance, thickness of the boundary layer, and transition time). In parallel, the limiting current density (LCD) of the ED stack was determined by the Cowan and Brown method [28]. Briefly, whey, NaCl, and Na_2_SO_4_ were circulated in the ED stack (Figure 1) as for the main experiments. The voltage was gradually increased from 0 to 40 V by increments of 0.5 V, and the resulting current intensity was monitored. The values of intensity and voltage obtained were plotted as resistance (U/I) as a function of reciprocal current (1/I). LCD was determined by the intersection of the tangents of the curves (Figure 2). Since the LCD of each studied ED stack was very close (Figure 2), the control ED experiments were carried out at a current density corresponding to 70% of this value.

This underlimiting current condition corresponds to a constant current of 0.7 A (current density of 7 mA/cm^2^) and was applied during 1 h with no temperature control and at flow rates of 400 mL/min in whey and NaCl compartments. A volume of 10 mL of samples was collected every 15 min during the process. They were frozen at −30 °C until further analyses such as lactic acid, lactose, and mineral contents. To complete this protocol, both configurations were carried out in an overlimiting current condition at 3.0 A (corresponding to 30 mA/cm^2^, largely over the limiting current value determined) during 30 min with no temperature control and the same flow rates as in the underlimiting conditions. In this study, the temperature was deliberately not controlled. Indeed, since the overlimiting current would increase the temperature, by controlling the temperature increase, its potential effect on the molecules’ transfer and energy efficiency would have not been taken into account. Furthermore, in a context of sustainability, controlling the temperature increase during ED at the overlimiting temperature would use lots of energy, and the potential energy saving related to the increase in temperature would be lost. So, to test both processes in real conditions and to be more close to the reality, the temperature was consequently not controlled. The ED duration was fixed to 30 min since during preliminary tests, it was observed, as expected, that the demineralization was faster and did not need to reach 1 h as for the underlimiting conditions. A volume of 10 mL of samples was also collected at 0, 5, 10, 20 and 30 min. Then, they were frozen until further analyses. All ED combinations of current conditions and configurations were repeated three times.

### 2.4. Analysis Methods

#### 2.4.1. Membrane Electrochemical Characteristics

Current–voltage (CV) curves as well as chronopotentiograms (ChP) were determined according to Villeneuve et al. [29] and Mikhaylin et al. [22]. Briefly, AEM and NF membranes were placed between two Haber–Luggin’s capillaries with an external diameter of 0.8 mm. A 0.02 M NaCl solution was circulated at flow rates of 32.9 mL/min for AEM and 32.4 mL/min for NFX. The current density, resistance, and thickness of the boundary layer were determined according to the CV curves, while the transition time representing the time required to reach the limiting current was determined according to the ChP.

#### 2.4.2. pH

The pH of both compartments (NaCl and whey) was measured using a Symphony pHmeter (Model SP70P, VWR, West Chester, PA, USA) equipped with a temperature probe to compensate for temperature changes.

#### 2.4.3. Lactic Acid and Lactose Contents

Lactic acid and lactose concentrations in collected samples during ED were determined with high-performance liquid chromatography (HPLC) with a Waters chromatograph (Waters Corp., Milford, MA, USA) equipped with a Hitachi differential refractometer detector L-7490 (Foster City, CA, USA), a 600E controller, a column oven, and a thermostated 717 Plus autosampler. An ICSep ICE-ION-300 column (Transgenomic, Omaha, NE, USA) was used with 8.5 mM of H_2_SO_4_ (180 µL H_2_SO_4_/L) solution as the mobile phase and at a flow rate of 0.4 mL/min. The column temperature was kept constant at 40 °C. Samples were centrifuged during 5 min at 5000 rpm (Allegra^TM^ 25R Centrifuge, Beckman Coulter, Brea, CA, USA) and filtered (0.22 µm nylon; CHROMSPEC Syringe Filter; Chromatographic Specialties, Brockville, ON, Canada) before injection (15 µL). Each sample was analyzed during a 36 min run time. Lactose anhydrous (PHR1025) and L-(+)-lactic acid (L1750) from Sigma-Aldrich (Saint-Louis, MO, USA) were used as external standards.

The deacidification or lactic acid recovery rate (DaR) related to each treatment was calculated by considering the acid lactic concentration detected in the samples collected at the beginning (C_i_ in ppm) and the end (C_f_ in ppm) of ED treatments according to Equation (1).

(1) $$DaR=\left(1-\frac{C_{f}}{C_{i}}\right)\times 100$$

#### 2.4.4. Conductivity

Acid whey and NaCl conductivities were measured with an YSI conductivity meter (Model 3100, Yellow Springs Instrument, Yellow Springs, OH, USA) equipped with an immersion probe (Model 3252, cell constant K = 1.0/cm). Then, the demineralization/mineralization rate based on the conductivity measurement was calculated with the same equation than for the deacidification rate (Equation (1)), considering the conductivity of whey and NaCl at the beginning (C_i_ in mS/cm) and the end (C_f_ in mS/cm) of each treatment.

#### 2.4.5. Mineral Content

As described by Dufton et al. [27], the samples collected during ED treatment were thawed at 4 °C before their dilution 1:20 in Milli- Q water to reach a final volume of 10 mL. Calcium, potassium, magnesium, sodium, and phosphorus concentrations were determined by optical emission spectrometry with inductively coupled plasma as an atomization and excitation source (ICP-OES Agilent 5110 SVDV Agilent Technologies, Victoria, Australia), using the following wavelengths (nm): 393.366; 396.847; 422.673 (Ca), 766.491 (K), 279.553; 280.270; 285.213 (Mg), 588.995; 589.592 (Na), 177.434; 178.222; 213.618; 214.914 (P). The analyses for all ions were carried out in axial and/or radial view. The demineralization rate based on the mineral concentration for each treatment was calculated using Equation (1) while considering the total ion concentration in acid whey at the beginning (C_i_ in ppm) and at the end (C_f_ in ppm) of each treatment.

#### 2.4.6. Overall System Resistance

The voltage (U) and current intensity (I) directly obtained from the two power supplies (Model 9110, BK Precision, Yorba Linda, CA, USA and Ionics Inc, Watertown, MA, USA) were used to calculate the global system resistance (R) according to Ohm’s law (U = R·I).

#### 2.4.7. Energy Consumption

The energy consumption (in Wh) was calculated according to Equation (2) where I is the current intensity (in A), U(t) tis he voltage (in V) as a function of time, and t is the duration of the ED treatment (min.)

(2) $$\mathrm{EC}\;\left(\mathrm{Wh}\right)=\frac{I\int U\left(t\right)\mathrm{dt}}{60}$$

Energy consumption for demineralization was calculated according to the concentration of K^+^ migrated from whey, since it is the ion having the higher migration rate [27].

### 2.5. Statistical Analyses

Data were subjected to one-way and two-way, and three-way analyses of variance (ANOVA). A Tukey multiple comparison test was performed to compare treatments together at a probability level of *p* < 0.05 (SAS software, version 6.3 for Windows, SAS Institute, Inc., Cary, NC, USA).

## 3. Results and Discussion

### 3.1. Electrochemical Characterization of Membranes

**Figure 3:**
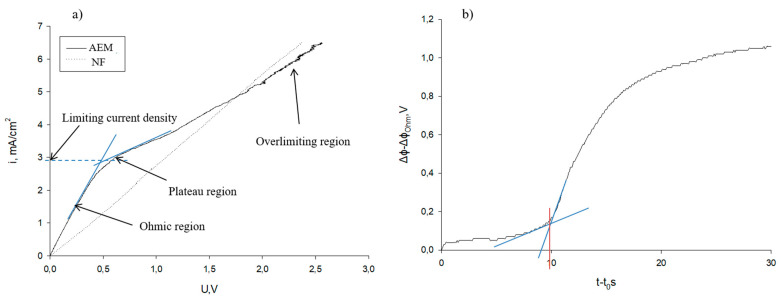
Representative (**a**) current–voltage (CV) curves for AEM and NF membranes and (**b**) ChP for an AEM membrane at current density of 2.9 mA/cm^2^.

CV curves were analyzed for both AEM and NF membranes. The experimental values of LCD were determined by the intersection of the extrapolated tangential lines of the Ohmic and plateau regions [29]. For the NF membrane, only the Ohmic phase was recorded in the range of 0–2.5 V (Figure 3a), which indicated that there was no limiting current in the voltage range analyzed (a complementary test was performed in the range of 0–5 V, still confirming the absence of a limiting current). In addition, the straight curve demonstrated that the NF membrane does not selectively separate ions from NaCl solution, since they all migrated through the membrane. For the AEM, a limiting current was observed, as the three distinct regions of CV curve were recorded (Figure 3a). Indeed, theoretical and experimental LCDs were 3.5 ± 0.1 and 2.9 ± 0.3 mA/cm^2^, respectively. These results were in accordance with Villeneuve et al. [29] for the same membrane. The fact that the NF membrane did not show any LCD explained why the values of LCD, determined previously by the Cowan and Brown method (see Section 2.3), were quite the same for both configurations of ED whatever AEM or NF membranes were stacked in the cell. In addition, the LCD of the EDNF stack depends mostly on LCD on CEM and the conductivity of the diluate solution.

Figure 3b illustrates the ChP measured at 2.9 mA/cm^2^ for the determination of the transition time, before the limiting current begins, for AEM, since no limiting current was observed for the NF membrane. The resistance, thickness of the boundary layer, and transition time were 44.4 ± 5.4 Ω, 284 ± 26 μm, and 11.1 ± 0.3 s, respectively These results were in accordance with those obtained by Villeneuve et al. [29] for the same membrane.

### 3.2. pH Evolution

**Figure 4:**
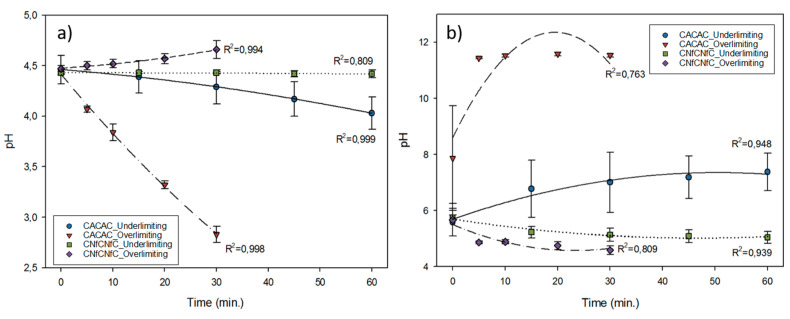
pH evolution in (**a**) whey and (**b**) NaCl solution during electrodialysis (ED) according to the configurations (CACAC and CNfCNf) and the current conditions (underlimiting and overlimiting) used.

The pH evolution in the whey compartment was influenced by the current condition applied and the membrane configuration tested (*P* < 0.001 for both). Indeed, for the CNfCNfC configuration, the pH was stable at 4.5 during the treatments in the underlimiting current condition, while it increased from 4.47 ± 0.03 to 4.66 ± 0.09 in the overlimiting one (*P* < 0.001) (Figure 4a). For the CACAC configuration, the pH of whey decreased differently for both current conditions (*P* < 0.001); in the underlimiting current condition, it decreased from 4.46 ± 0.14 to 4.03 ± 0.16, while the decrease in the overlimiting condition was more pronounced with a final value of 2.83 ± 0.08 (Figure 4a). For the NaCl compartment (Figure 4b), no statistical difference was observed for all tested current conditions (*P* = 0.242), but a general trend of decrease in pH was observed. Only the membrane configuration has an impact on pH evolution for each treatment (*P* < 0.001). Indeed, both CACAC underlimiting and overlimiting current conditions showed an increase in pH from 5.58 ± 0.49 to 7.37 ± 0.67 and 7.86 ± 1.87 to 11.53 ± 0.06. The opposite trend was observed for CNfCNf, since the pH decreased for both current conditions, until the end of the process, from 5.74 ± 0.09 to 5.03 ± 0.21 and from 5.66 ± 0.58 to 4.58 ± 0.15 in underlimiting and overlimiting conditions, respectively.

As previously mentioned in the literature for the CACAC configuration [26,27], the pH decrease in the whey compartment might be due to the water-splitting phenomena related to reaching LCD, which leads to proton electrogeneration at the diluate side of AEM. This effect was more intense for the overlimiting current condition on this configuration, meaning that the LCD of AEM has truly been surpassed, although water-splitting phenomenon still probably occurred at the CEM. Similar results were observed and explained by Lemay et al. [30] in the case of sweet whey. According to these authors, the pH variation is explained by two phenomena; firstly, the dissociation of singly charged ampholyte anions which occurs as they cross the membrane, leading to the release of protons in the depleted solution, and secondly, the water dissociation catalyzed by ionogenic groups of AEMs and by weak-acid anions. Moreover, these authors mentioned that with the increase in current density, the pH of the AEM internal solution in the layer adjacent to the diluate side increases and with this increase in pH, the concentration of doubly charged anion of weak acid increases in this interfacial layer. Consequently, a singly charged anion, which crosses the membrane, enters the solution of higher pH value and is then transformed into a doubly charged anion. Then, the protons are released in the whey solution, decreasing its pH, and in a more pronounced way in the overlimiting condition, since the current density is higher and more weak acid anions migrated [30,31,32]. For the CNfCNfC configuration, the pH in the underlimiting current condition remained stable due to the buffering capacity of whey, as previously explained. However, the pH in overlimiting conditions increased during the treatment, and it can be explained by the same water splitting phenomenon than for the CACAC configuration but only at the interface of the CEM. It was the first time that the pH evolution of a solution treated by ED with a nanofiltration membrane is reported.

### 3.3. Deacidification and Lactic Acid Migration

**Figure 5:**
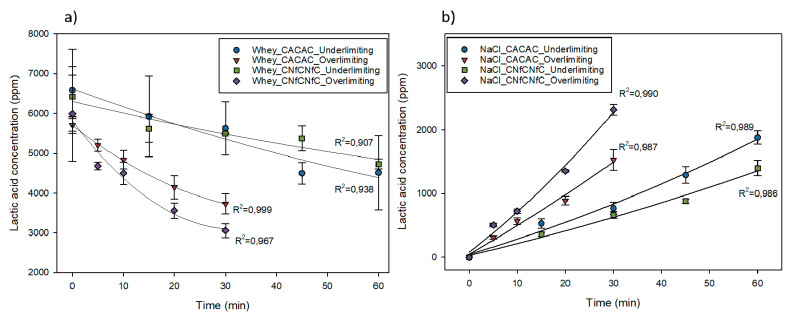
Evolution of lactic acid in (**a**) whey and (**b**) NaCl compartments during ED according to the configurations (CACAC and CNfCNf) and the current conditions (underlimiting and overlimiting) used.

As shown in Figure 5, in the whey compartment, the concentration of lactic acid decreased according to the membrane configuration and the current condition. However, there was no statistical difference between treatments (*P* = 0.595 for configuration, *P* = 0.516 for current, *P* = 0.314 for double interaction configuration/current). In contrary, considering the NaCl compartment, it was confirmed that lactic acid migrated from the whey compartment to the NaCl compartment and was influenced by the double interaction between the membrane configuration and current condition (*P* < 0.001). Furthermore, in the underlimiting current condition, the CACAC configuration led to a greater migration of lactic acid anion while its migration was higher in the overlimiting condition for the CNfCNfC configuration. Indeed, this organic compound was present mostly in a form of lactate (around 75%) at the pH of raw acid whey (4.45 ± 0.02). Consequently, it was able to migrate through the positively charged membranes (AEM and NF), whatever the configuration.

These results of lactate migration were confirmed by the deacidification rates calculated for each treatment. However, only the current condition had an impact on the global deacidification rate (*P* = 0.027). As shown in Table 2, the overlimiting current condition had a greater impact on the deacidification rate than the underlimiting current condition. This effect was more considerable on the CNfCNfC configuration (*P* = 0.020) between the two current conditions. Indeed, after only 30 min, the deacidification rate obtained in the overlimiting condition (40.00% versus 26.03%) had practically doubled compared to the underlimiting condition after 60 min of treatment. Consequently, the replacement of AEMs by NFs in an ED stack with the application of an overlimiting current condition could lead to a higher deacidification rate.

The results obtained for the CACAC configuration showed that the migration of lactate was not enhanced or improved by the overlimiting condition and the formation of EVs. Indeed, EVs are known to shrink the laminar boundary layers as well as to reduce or eliminate the concentration polarization at the membrane interface, leading to the release of current carriers and a better mass transfer of ionic species [22,33,34,35]. However, all the studies reported in the literature were carried out on model salt solutions, and solutions containing organic acids had never been tested. Thus, it appeared from these results that the mass transfer of organic acid seemed to be not improved by electroconvective vortices in contrary of mineral species.

Opposite to the CACAC configuration, for the CNfCNfC configuration, the recovery of lactic acid was almost doubled in the overlimiting condition. Different hypotheses can be proposed to explain these results; the pore size of the NF membrane, the temperature evolution during the process, and the fact that no LCD was obtained for NF membrane. Indeed, the higher pore size of NF membrane, in comparison with an IEM, can explain the better deacidification rate obtained. Furthermore, as reported in the literature, NF membranes have a high permeability for monovalent cations and organic acid anions with low molecular weight, but they limit the passage of organic compounds with a molecular mass that exceed 300 Da (ex. lactose) [36,37]. They also allow a greater permeability for all species when the system is reaching higher temperature because of the expansion of the polymer included in their structure [8]. In this case, the temperature considerably increased during each treatment (20 to 33 °C in underlimiting and 22 to 51 °C in overlimiting condition), because it was not controlled. Indeed, the increase in temperature is a potential advantage of the overlimiting condition, since it will improve the transfer of ions and in a context of sustainability, it could lead to potential energy saving. This increase in temperature might be due to the Joule effect or the continuous function of pumps, and it might have led to a greater mass transfer with nanofiltration membranes. Finally, the fact that no LCD was obtained for this type of membrane could also contribute to the better migration of organic acid anions. Indeed, the production of H^+^, in the whey compartment at the interface of the AEM after the LCD was reached would have impacted the migration of the lactate due to its pKa of 3.86. The H^+^ produced would have reacted with some lactate anions to produce non-charged lactic acid, decreasing their potential to migrate. Indeed, Serre et al. demonstrated that the production of OH^−^ during the deacidification of cranberry juice by ED with a bipolar membrane increased the concentration of dissociated, and consequently charged, organic acids according to their pKa. The opposite applied in the case of acid whey [38].

Recently, Talebi et al. used the combination of diafiltration (Dia)-NF and ED for the removal of lactic acid in separated steps. The use of Dia-NF prior to ED resulted in a 71% removal of lactic acid, compared to 36% with NF only prior to ED. In the present study, 40.00 ± 4.33% were obtained with CNfCNf configuration in the overlimiting current in only one step [39]. Indeed, the use of the NF membrane in an ED cell for acid whey treatment has never been performed before and demonstrated interesting results in terms of lactic acid recovery.

### 3.4. Lactose Evolution

The concentration of lactose remained constant in the whey compartment during the whole process for each treatment condition (results not presented here). No lactose was found in the NaCl compartment whatever the configuration used or the current condition applied. These results showed that CACAC and CNfCNfC membrane configurations allowed the deacidification of acid whey while preserving its compounds of interest such as lactose. Moreover, as reported by Chandrapala et al. (2016) [8], assuming the fact that lactose has no charge, it can only pass through a membrane by sieving or membrane size exclusion. Hence, the results demonstrated that an AEM or a 500 Da cut-off NF does not allow the migration of lactose. Similar results were obtained by Dufton et al. [27] and Chen et al. [26] on simultaneous acid whey demineralization and lactic acid recovery by conventional ED using a CACAC configuration. For CNfCNf configuration, it was performed for the first time in this study.

### 3.5. Conductivity Evolution and Demineralisation

**Figure 6:**
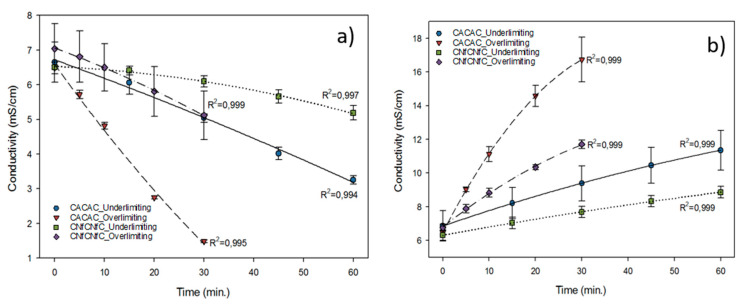
(**a**) Whey and (**b**) NaCl conductivity during ED for all configurations and current conditions tested.

The configuration (*P* < 0.001 for both whey and NaCl compartments) and the current condition applied (*P* < 0.001 for both whey and NaCl compartments) had an impact on the evolution of conductivity during treatments (*P* = 0.044 and *P* < 0.001 respectively for whey and NaCl compartments) (Figure 6). In the whey compartment, whatever the current condition, the decrease in conductivity was higher for CACAC than CNfCNfC, which corresponded to demineralization rates of 50.82% versus 20.32% in underlimiting and 77.23% versus 27.51% in overlimiting current modes (Table 3). Moreover, the decrease in conductivity measured in the whey compartment (Figure 6a) was higher in overlimiting than in underlimiting conditions. The same trend was observed in the NaCl compartment but in terms of mineralization (Figure 6b). For demineralization, the CACAC configuration was more effective than CNfCNfC, whatever the current condition used, leading to higher conductivity decreases (Figure 6a) and higher demineralization rates (Table 3). Those results showed a significant effect of the combination of the configuration and the current applied on the demineralization rate (*P* = 0.001).

**Figure 7:**
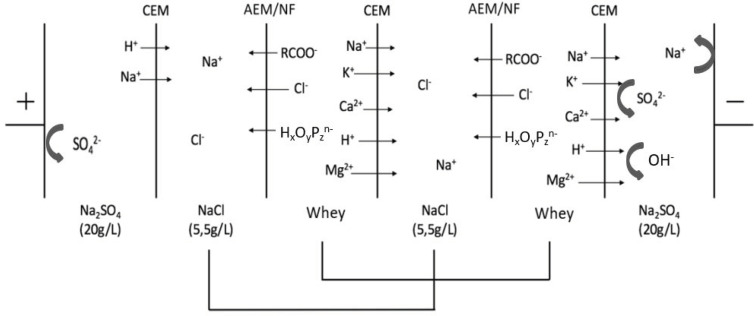
Scheme of ionic migration during ED treatments.

These conductivities and demineralization/mineralization rates can be explained by the migration of lactate and other ions through their respective membranes under the effect of the electric field applied. Hence, positively charged species (Na^+^, Ca^2+^…) migrated through the CEM, while negatively charged species (lactate, citrate, Cl^−^, H_x_P_y_O_z_^n−^) migrated through the AEM or NF membrane. By leaving the whey compartment, those species contributed to the decrease in its conductivity, which led to an increase in the demineralization rate and to a simultaneous increase of the conductivity in the NaCl compartment (Figure 7).

As also shown in Figure 6, a logical demineralization of whey and mineralization of NaCl were observed in the whey and NaCl compartments. While the conductivity decreased in the whey compartment, it increased more in the NaCl compartment. This behavior can be related to the buffering capacity of whey deriving from its protein content, organic acids and minerals such as inorganic phosphates [40]. According to the pH, those compounds can react with protons or hydroxide ions generated by the water dissociation at the membrane interface and lead to an overestimation of conductivity variation [30,41]. Lin Teng Shee et al. [42] demonstrated for the demineralization of whey by ED with bipolar membrane that the conductivity was underestimated by nearly 50% the experimental demineralization rates. Since the NaCl compartment had a lower buffering capacity at the beginning of the process than the whey one, the influence of protons on its conductivity was more easily noticeable. This result was confirmed by the higher demineralization rates calculated with the total cations concentrations, also demonstrating that the generation of protons had an impact on the conductivity measured. This effect was more significant in the overlimiting current condition where the calculation with total cations leads to 10 units of percentage of higher demineralization rates than the one calculated with conductivity.

In Dufton et al., 64% of demineralization was reached after 180 min with the CACAC configuration, mainly in the underlimiting condition. In the present study, a higher demineralization rate (77.2%) was obtained in 30 min in the overlimiting current [41]. These results confirmed that in the overlimiting current condition, EVs generated at the AEM can lead to a better demineralization of acid whey, as presumed in the literature [22,33]. However, the current condition did not have a strong effect as for the deacidification on the CNfCNfC membrane configuration. Indeed, in terms of demineralization, since the NF membrane is not selective to mineral species and does not reach any LCD, as demonstrated previously by the electrochemical characterization (see Section 2.3), its efficiency in mineral selectivity and increase in mass transfer due to EVs does not apply for this membrane. This fact explains the lower demineralization efficiency in both conditions of current for the CNfCNfC membrane configuration.

### 3.6. Ion Migration

**Figure 8:**
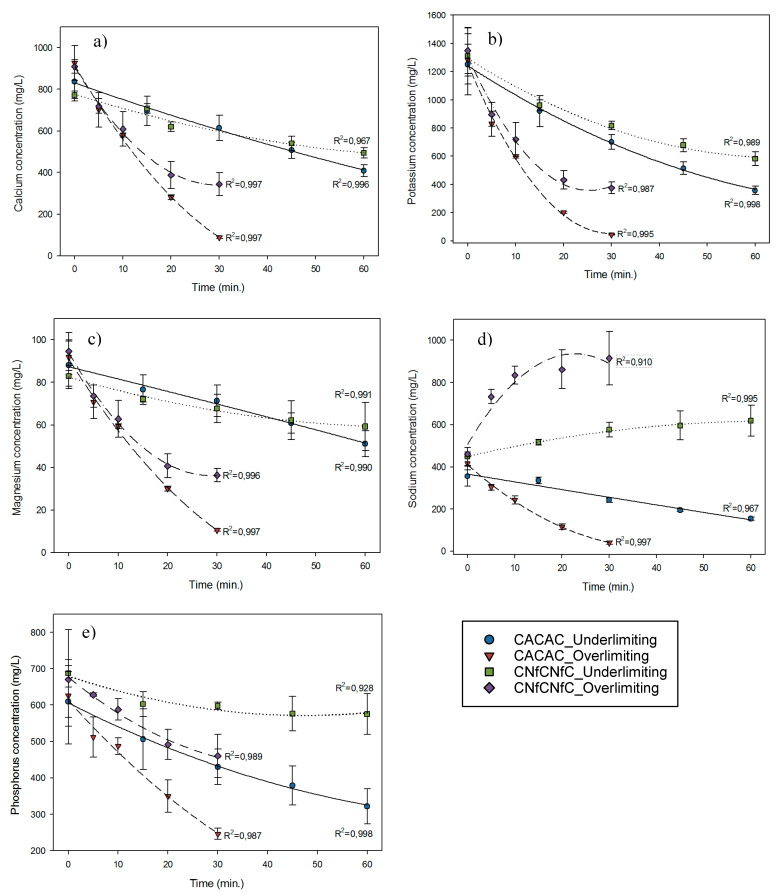
Ion concentration in whey compartment during ED treatments: (**a**) calcium, (**b**) potassium, (**c**) magnesium, (**d**) sodium, and (**e**) phosphorous.

Figure 8 and Table 4 represent the concentration of different cations and anions migrated from acid whey to NaCl and the ion migration rate at the end of ED treatments. Calcium migration was influenced by a double interaction between the membrane configuration and the current condition applied (*P* = 0.042). Indeed, the current applied in the overlimiting condition increased the migration rates obtained for the CACAC (*P* < 0.001) and CNfCNfC (*P* = 0.0003) configurations. However, the configuration had no impact on the demineralization rates obtained in the underlimiting current condition (*P* = 0.093), since it only had an impact while the overlimiting current condition was applied (*P* < 0.001). The same double interaction was observed for sodium migration (*P* < 0.001), but it has opposite origins. For this ion, the membrane configuration influenced drastically the migration rates whatever the current condition applied (*P* < 0.001 for the underlimiting and overlimiting currents). The current condition had only a statistical impact on the CACAC membrane configuration (*P* < 0.001), and it did not occur on the CNfCNfC membrane configuration (*P* = 0.259) because of the large standard deviations obtained for the three repetitions carried out on CNfCNfC in the overlimiting condition. These results for sodium were also explained by the CV curve, which indicated that the NF membrane allowed its migration. For potassium ion, its migration was influenced by the distinct effect of the configuration and current condition (*P* = 0.207). Each result was different depending on the membrane configuration used (*P* = 0.001) and the current applied (*P* = 0.003). The same trend was observed for magnesium and phosphorus (only anion analyzed), since configuration (*P* = 0.005 and *P* < 0.001 respectively) and current (*P* < 0.001 and *P* = 0.007 respectively) alone had separate effects (*P* = 0.186 and *P* = 0.776 for double interaction respectively) on their migration.

More generally, whatever the ED configuration or condition of current applied, the potassium was the ion having the highest migration rate, and it can be due to its high electrophoretic mobility and concentration [27]. As already reported in the literature, when the potassium concentration decreased, the migration of other cations is more intense [27,30]. Moreover, as mentioned in Dufton et al. [41], a low current density can lead to the migration of divalent ions when the solution is depleted in potassium due to their stronger interactions with the membrane functional groups, while a higher current density favors the concentration polarization and increases the migration of monovalent ions, which is related to their higher diffusivity in the membrane’s boundary layer [41,43,44]. This explains why calcium and magnesium closely followed potassium with similar migration rates for every configuration in the overlimiting condition. However, in the underlimiting condition, magnesium had a smaller migration rate (41.43 ± 9.34%) that can be explained by its slower mass transfer [45] and the absence of EVs that can enhance its migration and all other cations [22,33].

Regarding the phosphorous-containing ion migration, one can observe that H_x_O_y_P_z_^n−^ were anions having lower migration through the AEM and NF membrane compared to cation migration through the CEM in all the conditions tested. This fact is due to the relatively low electrophoretic mobility of these anions compared to the monovalent chloride and other organic anions. Moreover, the phosphorous-containing ionic migration is hampered by competitive anions having higher concentration in the whey solution (e.g., chloride, lactate). Furthermore, as shown in Figure 8 and by the negative sign in Table 4, the concentration of sodium increased in the whey compartment during ED treatment in the CNfCNfC configuration. This means that sodium cations competed with anions, including phosphorous-containing ones, to migrate through the NF membrane having low selectivity, as was discussed above.

### 3.7. Overall System Resistance

**Figure 9:**
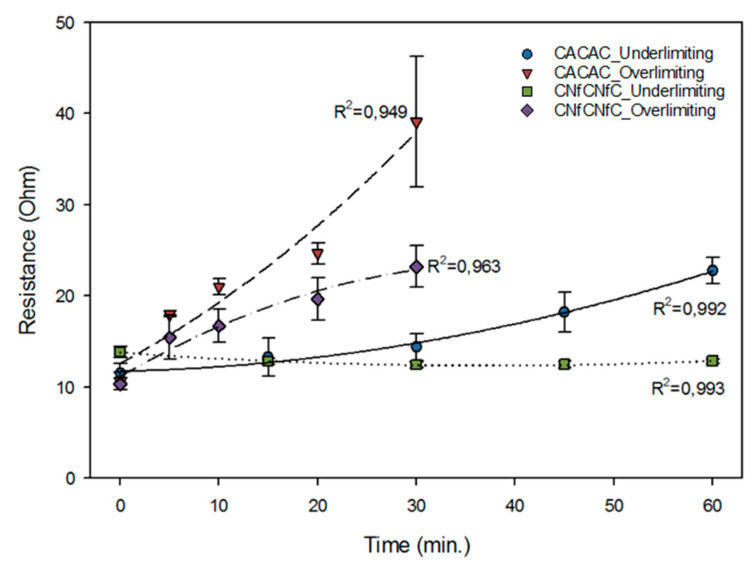
Overall system resistance of the electrodialysis cell.

There was a double interaction between the membrane configuration and current condition applied on the overall system resistance measured during ED treatments (*P* < 0.001). Indeed, the application of the overlimiting condition increased the cell resistance in comparison with the underlimiting one, whatever the configuration. However, in the underlimiting condition, the overall system resistance of CNfCNfC remained stable all along the treatment, while there was an increase in the resistance of CACAC (Figure 9). However, this increase of resistance is largely lower that in the overlimiting condition (28.46 versus 11.29 Ω).

The overall system resistance mostly depended on solutions and membrane resistances. Initially, NaCl and whey had similar conductivity values (Figure 8). However, as ED treatments progressed, the whey compartment became demineralized, and its resistance increased, since less and less ions were available to carry the current through the membranes. The application of the overlimiting current condition promoted this effect. This was confirmed by results previously presented in Table 4, when CACAC in the overlimiting condition led to the highest demineralization rates for every ion, and it was also the configuration at which the overall system resistance increased the most. The opposite trend could be observed for CNfCNfC in the underlimiting condition having the lowest ion migration rates and a stable overall resistance during the whole ED treatment. The stable global resistance in the CNfCNfC configuration is related to the fact that the NF membrane has a lower selectivity to cations: mainly sodium has migrated through this membrane, as demonstrated in Table 3, and with electrochemical characterization of the NF membrane.

### 3.8. Energy Consumption

There was no statistical difference in energy consumption for the deacidification step with both membrane configurations when the underlimiting current condition was applied (Table 5), leading to the conclusion that both ED stacks needed the same energy for the migration of the same quantity of lactic acid recovered, as previously presented in Table 2. In overlimiting current conditions, more energy was consumed than in underlimiting ones as expected, since 3A was applied instead of 0.7A: the increase in current was 4.3-fold, but the energy consumption increased respectively by 11 and 8-fold for CACAC and CNfCNfC configurations. Membrane configuration and current condition had an impact on results at overlimiting currents (*P* = 0.005). A larger amount of energy was necessary to deacidify acid whey when using the CACAC configuration compared to the CNfCNfC configuration. Indeed, a conventional stack needed around 68% more energy than the stack with the NF membrane. This energy cost was not justified, since this configuration led to a lower deacidification rate, as presented in Table 2. In this case, the CNfCNfC configuration and NF membrane seemed more interesting in terms of deacidification if an overlimiting current condition was applied.

As reported by Chen et al. [26], a standard CACAC configuration consumed 4 to 9 kWh/kg of lactate migrated. These results coincide with the ones presented in Table 5. Consequently, the CNfCNfC configuration can be as efficient as a CACAC configuration when the underlimiting condition is applied.

Concerning the demineralization step, energy consumption, as presented in Table 5, followed the same trend as deacidification, since similar energy consumptions (around 20 Wh/g K^+^) were observed for both configurations. However the overlimiting condition increased by 7–9 times the energy consumption compared to the underlimiting condition, and these results are not in accordance with Dufton et al., since the use of pulsed electric fields in their study allowed a decrease in energy consumption compared to the constant current in the underlimiting condition [35]. Additionally, Bazinet and Moalic [24] reported that the use of an NF membrane in an ED stack for the demineralization of sea water significantly increased the level of energy required by the process.

In addition, it seemed that the demineralization of acid whey consumed more energy than for deacidification. Indeed, in the underlimiting condition, the energy consumption had practically doubled for both membrane configurations to demineralize acid whey (*P* < 0.001), meaning that the difference of potential applied had a greater impact on minerals than on lactate ions. However, even if the CACAC configuration needed more energy in the overlimiting current condition, it led to greater demineralization rates than all the other tested conditions. As for the CNfCNfC membrane configuration, it required the same amount of energy than the CACAC, but for half the demineralization rate obtained. Consequently, even if this configuration was promising for deacidification, it was less interesting for demineralization.

It is also important to mention that a larger-scale system can diminish the energy consumption. If the overlimiting condition is selected, a larger quantity of acid whey can be deacidified and demineralized in a shorter time. The investment depends on which component remains more rentable for the industrial.

## 4. Conclusions

It was the first time that NF was used as an AEM in an ED stack for the treatment of acid whey and compared with a classic AEM-containing ED stack while applying conventional underlimiting and emerging overlimiting current conditions. The results showed that the overlimiting condition led to greater deacidification rates with CNfCNfC configuration (40%) while it had a more significant impact on demineralization with a CACAC configuration (87%). However, these two optimal conditions needed more energy than treatments performed with the underlimiting current condition. Their conductivity, pH, and global resistance were also more influenced by the water splitting phenomena that occurred while the limiting current was reached. Furthermore, it appeared that the selectivity for sodium was drastically decreased or suppressed in the overlimiting condition for the NF membrane, while no change was observed for other cations.

Further experiments also have to be carried out on the CNfCNfC membrane configuration to study the generation of EVs and their development on both AEM and NF membrane types. In addition, applying a pulsed electric field in both underlimiting and overlimiting current conditions could be interesting in order to compare their impact on acid whey treatment efficiency and to potentially enhance the demineralization and deacidification processes.

## Figures and Tables

**Table 1:** Composition and physicochemical characteristics of raw acid whey.

Analysis	Units	Raw Acid Whey
Total solid	g/L	42.50 ± 2.90
Total protein	g/L	7.15 ± 0.41
Lactose	g/L	30.48 ± 1.42
Lactic acid	g/L	6.18 ± 0.40
Ca	g/L	0.86 ± 0.07
Mg	g/L	0.09 ± 0.01
Na	g/L	0.42 ± 0.65
K	g/L	1.30 ± 0.04
P	g/L	0.65 ± 0.04
pH	-	4.45 ± 0.02
Conductivity	mS/cm	6.54 ± 0.45

**Table 2:** Deacidification rate of acid whey during ED for all configurations and current conditions tested.

Membrane Configuration	Current Condition	Deacidification	Time of Treatment
		%	min
CACAC	Underlimiting	31.66 ± 6.46 ^A^	60
	Overlimiting	34.70 ± 3.17 ^A^	30
CNfCNfC	Underlimiting	26.03 ± 4.93 ^A^	60
	Overlimiting	40.00 ± 4.33 ^B^	30

Values followed by different letters (A–B) are statistically different (*P* < 0.05), Tukey test.

**Table 3:** Demineralization rate of acid whey (in %) during ED for all configurations and current conditions tested.

Membrane Configuration		Demineralization Rate Based on Conductivity Measurement	Demineralization Rate Based on Total Cations Concentration (See Section 3.7)	Time of Treatment
		%	%	min
CACAC	Underlimiting	50.82 ± 5.48 ^C^	58.33 ± 2.59 ^C^	60
	Overlimiting	77.23 ± 0.96 ^D^	87.12 ± 1.51 ^D^	30
CNfCNfC	Underlimiting	20.32 ± 3.01 ^A^	23.40 ± 3.55 ^A^	60
	Overlimiting	27.51 ± 2.68 ^B^	41.10 ± 6.69 ^B^	30

Values followed by different letters (A–D) in a same column are statistically different (*p* < 0.05), Tukey test.

**Table 4:** Ion migration rates (g/100 mL of whey) from whey compartment at the end of ED for all configurations and current conditions tested.

Membrane Configuration	Current Condition	Ca	K	Mg	Na	P
		g/100 mL of Whey				
CACAC	Underlimiting	51.1 ± 2.1 ^A^	71.1 ± 3.6 ^C^	41.4 ± 9.3 ^B^	55.2 ± 3.1 ^B^	46.7 ± 3.4 ^C^
	Overlimiting	90.4 ± 1.4 ^C^	96.7 ± 0.5 ^D^	88.4 ± 1.6 ^D^	90.3 ± 2.5 ^C^	60.4 ± 4.4 ^D^
CNfCNfC	Underlimiting	41.3 ± 8.2 ^A^	54.9 ± 7.9 ^A^	28.9 ± 9.4 ^A^	−39.4 ± 28.1 ^A^	13.8 ± 3.3 ^A^
	Overlimiting	63.1 ± 9.2 ^B^	67.8 ± 13.5 ^B^	61.5 ± 9.5 ^C^	−69.2 ± 30.9 ^A^	29.6 ± 10.4 ^B^

Values followed by different letters (A–D) in a same column are statistically different (*p* < 0.05), Tukey test.

**Table 5:** Energy consumption during ED for all configurations and current conditions tested.

Membrane Configuration	Current Condition	Deacidification	Demineralization
		Wh/g Lactate Migrated from Whey	Wh/g K^+^ Migrated from Whey
CACAC	Underlimiting	9.29 ± 1.70 ^A^	19.78 ± 6.99 ^A^
	Overlimiting	109.48 ± 13.61 ^C^	174.41 ± 7.50 ^B^
CNfCNfC	Underlimiting	7.90 ± 1.50 ^A^	20.99 ± 1.72 ^A^
	Overlimiting	65.14 ± 13.55 ^B^	152.02 ± 27.62 ^B^

Values followed by different letters (A–B) in a same column are statistically different (*p* < 0.05), Tukey test.

## Abbreviations

AEM	anion-exchange membrane
CEM	cation-exchange membrane
CV	Current-Voltage
ED	electrodialysis
EDNF	ElectroDialysis with nanofiltration membrane
IEM	ion exchange membrane
EVs	electroconvective vortices
NF	nanofiltration
